# Genes ingenuity pathway analysis unveils smoothelin‐like 1 (SMTNL1) as a key regulatory protein involved in sodium pentobarbital‐induced growth inhibition in breast cancer

**DOI:** 10.1002/prp2.1153

**Published:** 2023-11-09

**Authors:** Bingwei Li, Xiaoyan Zhang, Xueting Liu, Ailing Li, Jianqun Han

**Affiliations:** ^1^ Institute of Microcirculation Chinese Academy of Medical Sciences & Peking Union Medical College Beijing China; ^2^ International Center of Microvascular Medicine Chinese Academy of Medical Sciences Beijing China

**Keywords:** breast cancer, ingenuity pathway analysis, LC–MS/MS, SMTNL1, sodium pentobarbital

## Abstract

We previously reported that sodium pentobarbital inhibited the growth of the breast cancer associated with the normalization of microcirculatory hemodynamics and oxygenation. Here, we aimed to screen the key regulatory proteins involved in pentobarbital‐induced normalization of microcirculatory hemodynamics in the breast cancer tissues. A nude mice model of xenograft was established using triple negative breast cancer cell line MDA‐MB‐231. After tumor cell implantation, the mice were subcutaneously injected with 50 mg/kg/day of sodium pentobarbital or an equal volume of solvent adjacent to the tumor for 14 days. Liquid chromatography linked to tandem mass spectrometry (LC–MS/MS) was used to analyze the difference in protein expression profile between the two groups. Ingenuity pathway analysis (IPA) was used to perform the canonical pathway analysis, upstream regulators analysis, and protein–protein interaction networks analysis. Screened proteins were confirmed by real‐time quantitative polymerase chain reaction (RT–qPCR) and Western blot analysis. A total of 101 differentially expressed proteins were revealed between groups. Canonical pathway analysis suggested that acute phase response signaling (*z* = 1, *p* = .00208), dilated cardiomyopathy signaling pathway (*z* = −2, *p* = .00671), and ILK signaling (*z* = 1, *p* = .0172) were key pathways with highlight associations. The mRNA and protein expressions of SMTNL1 were found significantly decreased in pentobarbital‐treated tumor tissues compared with those in controls (both *p* < .01). Nine important protein–protein interaction networks were identified, and of which, two contained multiple downstream regulatory proteins of SMTNL1. In conclusion, SMTNL1 is revealed as a key protein involved in pentobarbital‐induced growth inhibition signaling in breast cancer. SMTNL1 may become a new potential target for tumor microcirculation research.

## Graphical abstract

We previously reported that sodium pentobarbital inhibited the growth of breast cancer associated with the normalization of microcirculatory hemodynamics and oxygenation. Here we aimed to screen the key regulatory proteins involved in this process. Liquid chromatography linked to tandem mass spectrometry was used to analyze the difference in protein expression profile between two groups. Ingenuity pathway analysis was used to perform the canonical pathway analysis, upstream regulators analysis and protein‐protein interaction networks analysis. Nine important protein‐protein interaction networks were identified, and of which, two interaction networks (Figure A and B) with the key downstream proteins regulated by SMTNL1 are focused.

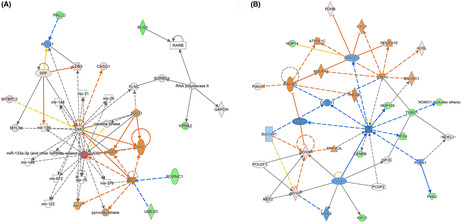

**Abbreviations**
cGMP: cyclic guanosine monophosphate
IPA: Ingenuity pathway
LC20: 20 kDa myosin regulatory light chain
LC–MS/MS: Liquid chromatography linked to tandem mass spectrometry
MLCK: myosin light chain kinase
MLCP: myosin light chain phosphatase
MYL: myosin light chain
NICR: National Infrastructure of Cell Line Resource
PKG: protein kinase G
RT‐qPCR: real‐time quantitative polymerase chain reaction
SMTNL1: smoothelin‐like 1
TNBC: triple‐negative breast cancer

## 1 INTRODUCTION

Breast cancer is the most common cancer accounting for more than 15% of new cancer cases in Chinese women and contributes to the most cancer deaths in the age group of 15–44 years.^1^ Adjuvant and neoadjuvant treatments have been proven to reduce the recurrence and mortality of breast cancer but may increase deaths from other causes including heart disease, leukemia, or other second cancers.^2^ Especially, triple‐negative breast cancer (TNBC), a highly heterogeneous disease, possesses a high incidence of metastases and poor prognosis.^3^ Multiple therapeutic regimens including poly ADP‐ribose polymerase inhibitors, immune‐checkpoint inhibitors, and antibody–drug conjugates have been established in recent years.^4^ However, chemotherapy resistance is more frequent to the established therapies in TNBC, and multiple chemotherapy response biomarkers are identified recently.^5^ Therefore, new optional drugs with potential anti‐TNBC effects are urgent to be explored.

Sodium pentobarbital is a commonly used anesthetic agent in animal experiments via its short‐acting sedative and respiration depressive effects.^6^, ^7^ Unexpectedly, phenobarbital shows an inhibitory effect on glioma development in an inducible rat model of neurogenic tumors.^8^ Xie et al.^9^ revealed that signaling pathways including ERK, c‐Jun MAPK, and PI3K/Akt were involved in the suppression of in vitro proliferation and migration of glioma cells by pentobarbital. Pentobarbital is also found to inhibit colon cancer growth and the incidence of liver metastases in a cancer cell‐injected mice model.^10^

Xenograft mouse models and genetically engineered models are commonly used to investigate pathological mechanisms of the breast cancer.^11^ Recently, we reported that pentobarbital inhibited breast cancer growth in mice bearing subcutaneous xenograft.^12^ This inhibitory effect is associated with the ability of pentobarbital to normalize the microcirculatory hemodynamics and oxygenation in tumor tissues. The perfusion of the capillary network is controlled by the vasomotor response of the arterioles.^13^ To unveil the key proteins regulating the microcirculatory hemodynamics involved in the antitumor effects of pentobarbital, we compared the differentially expressed proteins between pentobarbital‐treated tumor tissues and control tumor tissues and screened the key regulatory proteins using Ingenuity pathway analysis (IPA).

## 2 MATERIALS AND METHODS

### 2.1 Cell culture

The MDA–MB‐231 breast cancer cell line was purchased from the National Infrastructure of Cell Line Resource (NICR), Chinese Academy of Medical Sciences & Peking Union Medical College (China). The cells were then cultured in the Dulbecco's modified Eagle's medium (Thermo Fisher Scientific Inc., Waltham, MA) supplemented with 10% fetal bovine serum (Thermo Fisher Scientific Inc.). Cell culture was performed in a humidified incubator with 5% CO_2_ and maintained at 37°C.

### 2.2 In vivo tumor formation assay

Animal care and experimental protocols have been approved by the Institutional Animal Care and Use Committee at the Institute of Microcirculation, Chinese Academy of Medical Sciences (China, Approval No. WXH20210109). Xenograft model was established in eight 6‐week‐old female BALB/c nude mice, which were purchased from the SPF Biotechnology Co., Ltd. (Beijing, China). The mice were housed for 1 week before the model establishment. The mice were free to regular diet and water at 24 ± 1°C under a 12 h‐12 h light/dark cycle. Cell suspension containing 5 × 10^6^ MDA–MB‐231 cells in Matrigel (100 μL, BD Biosciences, San Jose, CA) was inoculated into the armpit of the nude mice. The mice were randomly divided into two groups (*n* = 4 in each group) 4 days after the implantation of the tumor cells. Mice in the treatment group were subcutaneously injected with 50 mg/kg/day of sodium pentobarbital (in 100 μL water, Sigma, St. Louis, MO) adjacent to the tumor for 14 days. Other mice injected with an equal volume of solvent served as controls. The volume of the tumors was calculated as the following formula: Volume = (length × width^2^)/2.

### 2.3 Protein digestion

Tumor tissues from each mouse were homogenized in the lysis buffer (8 M urea, 50 mM Tris, pH = 8.0). The proteins were extracted and then reduced with dithiothreitol (10 mM) and alkylated with iodoacetamide (55 mM). The proteins were transferred to an ultracentrifugal tube (10 KD) and buffered with 20 mM Tris HCl. Proteins were then digested using trypsin overnight at 37°C. The peptides were de‐salted and concentrated using a reversed‐phase (C18) cartridge. The solvent in elution products was removed by a vacuum centrifuge.

### 2.4 LC–MS/MS Analysis

The sample extracts were separated with an EASY‐n LC1000 UHPLC system (Thermo Scientific, Waltham, MA). Briefly, the column oven was kept at 60°C, and the samples were injected into a trap C18 column (5 μm, 75 μm × 2 cm), and then separated with a capillary LC C18 column (3 μm, 75 μm × 10 cm). The eluted duration was 60 min and the following gradient was applied: 6%–28% for 48 min and 28%–95% for 4 min buffer *B* (0.1% formic acid, 100% ACN). The flow rate was 0.6 mL/min. The eluted analytes were detected using an Orbitrap Fusion Lumos mass spectrometer (Thermo Scientific). Data‐independent acquisition (DIA) was performed following the parameters: Positive mode was used. One cycle contains one full scan with a maximum injection time of 54 ms, and 40 segment fragment scans. Full scan ranged from 350 to 1300 m/z and screened at 120 000 resolutions. Fragment spectra were collected at 3000 resolutions.

The raw data of DIA were analyzed with Spectronaut software (version 14.3, Biognosys, Schlieren, Switzerland). Raw files were searched against the SwissProt database. The samples were quantitative evaluation based on the MS2 area. Cross‐runs were normalized according to the global abundance area.

### 2.5 Real‐time quantitative polymerase chain reaction (RT‐qPCR)

Total RNA was collected from the tumor issues by using a Trizol reagent (Invitrogen, Carlsbad, CA). Total RNA at 1 μg was transcribed into cDNA using the HiFiScript cDNA Synthesis Kit (Cwbio, Beijing, China) in a total reaction volume of 15 μL. Then qRT–PCR was performed using SYBR FAST qPCR Master Mix Kit (Kapa Biosystems, Woburn, MA) on an ABI StepOne Plus Real–Time PCR System (Applied Biosystems, Foster City, CA). The sequences of primers were as follows: SMTNL1 forward 5′‐TGCCAATGACAGAGACAAGC‐3′ and reverse 5′‐TTGCATCAGCCTCCTCTTTT‐3′; MYL9 forward 5′‐TCTTCGCAATGTTTGACCAGT‐3′ and reverse 5′‐GTTGAAAGCCTCCTTAAACTCCT‐3′; GAPDH forward 5′‐ACAACTTTGGTATCGTGGAAGG‐3′ and reverse 5′‐GCCATCACGCCACAGTTTC‐3′. Real‐time PCR was performed for 3 min at 95°C, followed by 40 cycles of 3 s at 95°C and 20 s at 60°C. GAPDH served as internal control and each sample was analyzed in triplicates. The relative gene expression was determined by the 2^−ΔΔCt^ method.

### 2.6 Western blot analysis

Western blot analysis of tumor tissues from the treatment group or control group was performed by SDS–PAGE gel and ECL technique for signal detections (Amersham, Arlington Heights, IL). Tissue proteins were electrophoresed and then transferred to a PVDF membrane. The non‐specific reactivity was blocked with 5% nonfat dry milk and incubated with primary antibodies at 4°C overnight. Primary antibodies were anti‐SMTNL1 (sc‐390369, Santa Cruz Biotechnology, Santa Cruz, CA, 1:500 dilution), anti‐myosin light chain 9 (MYL9, ab64161, Abcam, Cambridge, MA, 1:1000 dilution), and anti‐β actin (ab8226, Abcam, 1:2000 dilution). The corresponding secondary antibodies were from Abcam and used at 1:10 000 dilution. The gray value was quantified using Image‐Pro Plus 6.0 software (Media Cybernetics, Bethesda, MD).

### 2.7 Statistical analysis

SPSS 21.0 version software (SPSS Inc., Chicago, IL) was used for statistical analysis. Data are presented as mean ± standard deviation (SD). Student's *t*‐test was used for a two‐group comparison. A *p*‐value <.05 was considered as statistical significance.

Proteins differentially expressed in pentobarbital‐treated tumor tissues as compared with control tumor tissues were determined by above or below the expression ratio of 1.5 and *p* ≤ .05 were considered significantly elevated or decreased. Then the proteins were investigated using IPA Analysis (Ingenuity Systems, Redwood City, CA). The canonical pathways, upstream regulators, and protein–protein interaction networks were analyzed to determine their biological significance. Fisher's exact test was used to calculate a *p*‐value in IPA analysis to determine the significance. In upstream regulators analysis, overlap *p*‐value and activation *z*‐score were both used to predict the regulatory relationship between upstream regulators and their targeted genes. *p*‐value <.05 and *z*‐score >2 or <2 were considered significant. In canonical pathway analysis, *p*‐value <.05 and *z*‐score ≠ 0 were considered significant. In network analysis, *p*‐value <.05 was considered significant.

## 3 RESULTS

### 3.1 Sodium pentobarbital suppresses the growth of breast cancer in vivo

**FIGURE 1:**
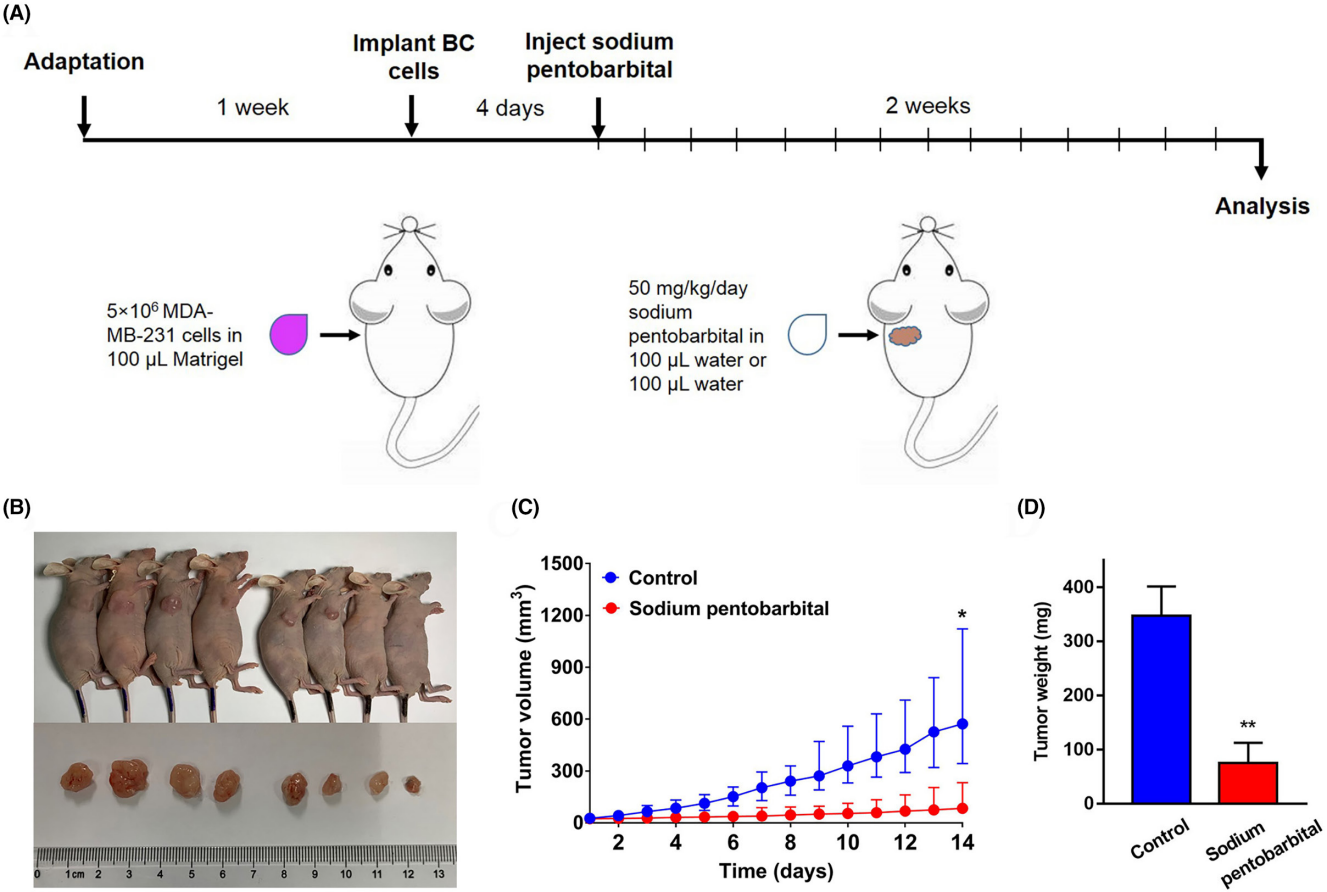
Sodium pentobarbital inhibits tumor growth in vivo in a mice xenograft model. (A) Schematic of the experimental design. Xenograft model was established in eight 6‐week old female BALB/c nude mice, which were housed for 1 week until cell suspension containing 5 × 10^6^ MDA–MB‐231 cells in 100 μL Matrigel were inoculated into the armpit of the mice. At 4 days after the implantation of the tumor cells, the mice in the treatment group were subcutaneously injected with 50 mg/kg/day of sodium pentobarbital (in 100 μL water) adjacent to the tumor for 14 days. Mice in the control group were injected with an equal volume of water. The tumor issues were used for subsequent analysis. (B) Morphologic characteristics of the xenograft tumors treated with or without sodium pentobarbital. (C) The tumor growth, expressed as the volume, was dynamically measured after treatment with or without sodium pentobarbital. (D) The weight of tumors was significantly decreased after the mice treated with sodium pentobarbital than that in controls. *n* = 4, *p < .05, **p < .01 vs. Control.

The schematic diagram of animal treatments was shown in Figure 1A. The mice were injected with or without sodium pentobarbital at 4 days after cancer cell implantation and the samples were analyzed 14 days later. The morphological features of xenograft tumors are shown in Figure 1B. The volume of tumors was significantly decreased in the mice treated with sodium pentobarbital compared with that in control mice (*p* < .05, Figure 1C). In addition, the weight of tumors was also significantly decreased after the mice treated with sodium pentobarbital than that in controls (*p* < .01, Figure 1D).

### 3.2 Analysis results of LC–MS/MS

**FIGURE 2:**
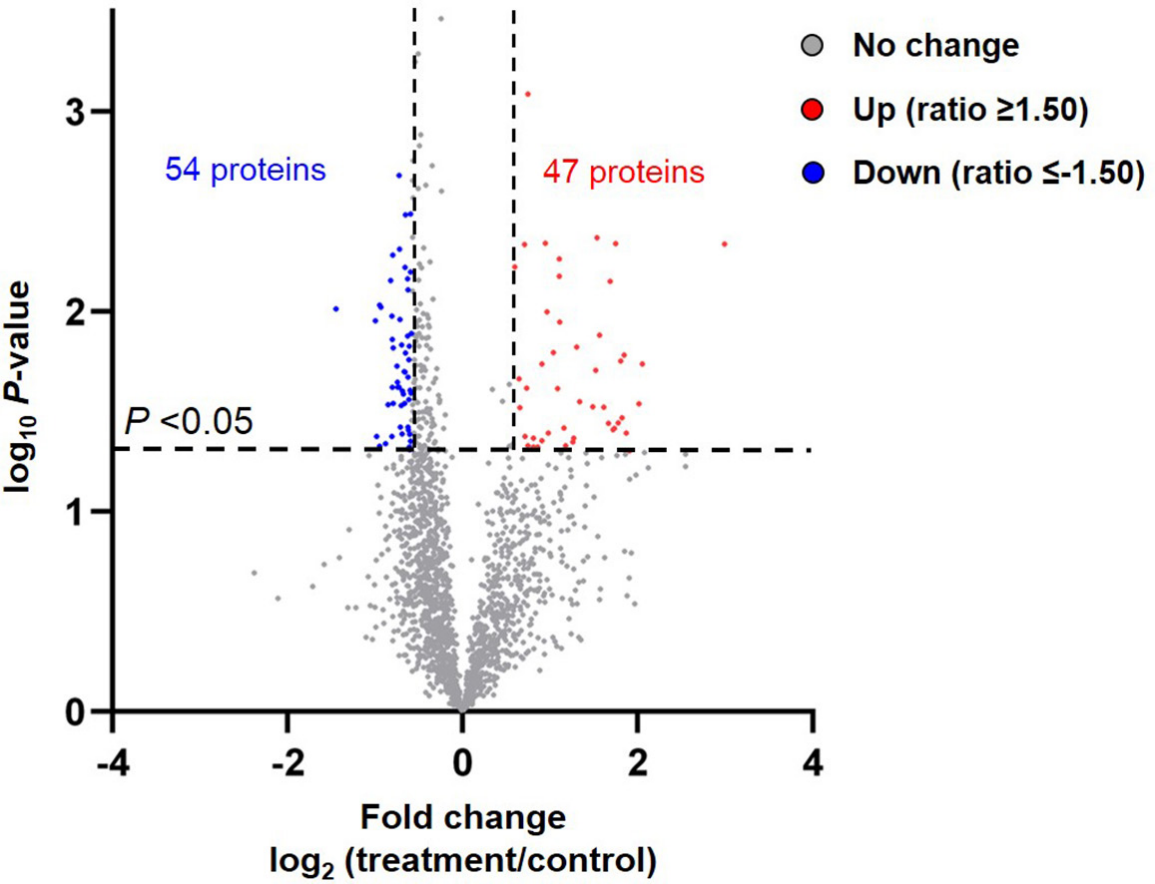
Differentially expressed proteins between sodium pentobarbital‐treated tumors and controls. This volcano plot indicates differentially expressed proteins with a ratio ≥ or ≤1.50 in the treatment group than that in the control group. Meanwhile, a *p*‐value <.05 was considered significant. *n* = 3.

The tumor samples collected from the sodium pentobarbital‐treated group and control group were analyzed by LC–MS/MS, and a total of 4102 proteins were quantified. Of these, 101 differentially expressed proteins were revealed between groups. A Volcano plot indicated that 47 proteins were differentially up‐regulated and 54 differentially down‐regulated in sodium pentobarbital‐treated tumors compared with controls (Figure 2). The top 25 differentially expressed proteins more abundant in sodium pentobarbital‐treated or control tumor tissues were shown in Tables 1 and 2, respectively. The protein accessions, gene symbols, protein descriptions, ratios, and the *p*‐value were summarized.

**TABLE 1:** Differentially expressed proteins are significantly more abundant in sodium pentobarbital‐treated tumor issues than in control tumor issues.

Protein accessions	Genes	Protein descriptions	Ratio (Treatment/control)	*p*‐value
P82350	*Sgca*	Alpha‐sarcoglycan	16.59	.034
Q8VC30	*Tkfc*	Triokinase/FMN cyclase	7.94	.005
Q9D783	*Klhl40*	Kelch‐like protein 40	4.15	.018
Q5XKE0	*Mybpc2*	Myosin‐binding protein C, fast‐type	4.03	.029
Q5SX40	*Myh1*	Myosin‐1	3.75	.050
A2ASS6	*Ttn*	Titin	3.65	.041
Q9JKS4	*Ldb3*	LIM domain‐binding protein 3	3.59	.017
O09165	*Casq1*	Calsequestrin‐1	3.53	.034
P05977	*Myl1*	Myosin light chain 1/3, skeletal muscle isoform	3.50	.018
A2AAJ9	*Obscn*	Obscurin	3.42	.036
Q62417	*Sorbs1*	Sorbin and SH3 domain‐containing protein 1	3.36	.005
Q5SX39	*Myh4*	Myosin‐4	3.33	.038
Q62234	*Myom1*	Myomesin‐1	3.29	.039
Q99PR8	*Hspb2*	Heat shock protein beta‐2	3.22	.007
Q7TQ48	*Srl*	Sarcalumenin	3.18	.036
A2ABU4	*Myom3*	Myomesin‐3	3.06	.030
P49817	*Cav1*	Caveolin‐1	2.96	.013
P62317	*Snrpd2*	Small nuclear ribonucleoprotein Sm D2	2.90	.004
P70402	*Mybph*	Myosin‐binding protein H	2.87	.020
P28650	*Adssl1*	Adenylosuccinate synthetase isozyme 1	2.80	.030
Q91ZA3	*Pcca*	Propionyl‐CoA carboxylase alpha chain, mitochondrial	2.53	.028
Q8VHX6	*Flnc*	Filamin‐C	2.47	.015
P54116	*Stom*	Erythrocyte band 7 integral membrane protein	2.41	.043
Q9Z1T2	*Thbs4*	Thrombospondin‐4	2.39	.045
Q6P8J7	*Ckmt2*	Creatine kinase S‐type, mitochondrial	2.26	.047

**TABLE 2:** Differentially expressed proteins are significantly more abundant in control tumor issues than sodium pentobarbital‐treated tumor issues.

Protein accessions	Genes	Protein descriptions	Ratio (Control/treatment)	*p*‐value
Q19LI2	*A1bg*	Alpha‐1B‐glycoprotein	2.73	.010
O35593	*Psmd14*	26S proteasome non‐ATPase regulatory subunit 14	1.99	.011
E9Q5K9	*Ythdc1*	YTH domain‐containing protein 1	1.98	.042
Q9WU56	*Pus1*	tRNA pseudouridine synthase A	1.93	.047
P52293	*Kpna2*	Importin subunit alpha‐1	1.93	.009
P59114	*Pcif1*	mRNA (2'‐O‐methyladenosine‐N(6)‐)‐methyltransferase	1.91	.010
Q9R0B9	*Plod2*	Procollagen‐lysine,2‐oxoglutarate 5‐dioxygenase 2	1.84	.046
P48428	*Tbca*	Tubulin‐specific chaperone A	1.81	.029
Q8BX10	*Pgam5*	Serine/threonine‐protein phosphatase PGAM5, mitochondrial	1.77	.007
Q6P9P6	*Kif11*	Kinesin‐like protein KIF11	1.75	.011
Q921D4	*Med6*	Mediator of RNA polymerase II transcription subunit 6	1.75	.042
Q9CWL8	*Ctnnbl1*	Beta‐catenin‐like protein 1	1.75	.024
Q9CR26	*Vta1*	Vacuolar protein sorting‐associated protein VTA1 homolog	1.74	.014
P80317	*Cct6a*	T‐complex protein 1 subunit zeta	1.74	.005
Q9ET54	*Palld*	Palladin	1.73	.029
Q9JJT0	*Rcl1*	RNA 3′‐terminal phosphate cyclase‐like protein	1.73	.015
Q6ZPJ3	*Ube2o*	(E3‐independent) E2 ubiquitin‐conjugating enzyme UBE2O	1.68	.019
Q8VI75	*Ipo4*	Importin‐4	1.68	.023
Q9QYI4	*Dnajb12*	DnaJ homolog subfamily B member 12	1.67	.024
Q689Z5	*Sbno1*	Protein strawberry notch homolog 1	1.66	.002
A2A4P0	*Dhx8*	ATP‐dependent RNA helicase DHX8	1.65	.024
Q64261	*Cdk6*	Cyclin‐dependent kinase 6	1.65	.005
P60229	*Eif3e*	Eukaryotic translation initiation factor 3 subunit E	1.64	.011
Q6P549	*Inppl1*	Phosphatidylinositol 3,4,5‐trisphosphate 5‐phosphatase 2	1.64	.038
Q8K224	*Nat10*	RNA cytidine acetyltransferase	1.63	.029

### 3.3 Canonical pathway analysis

**FIGURE 3:**
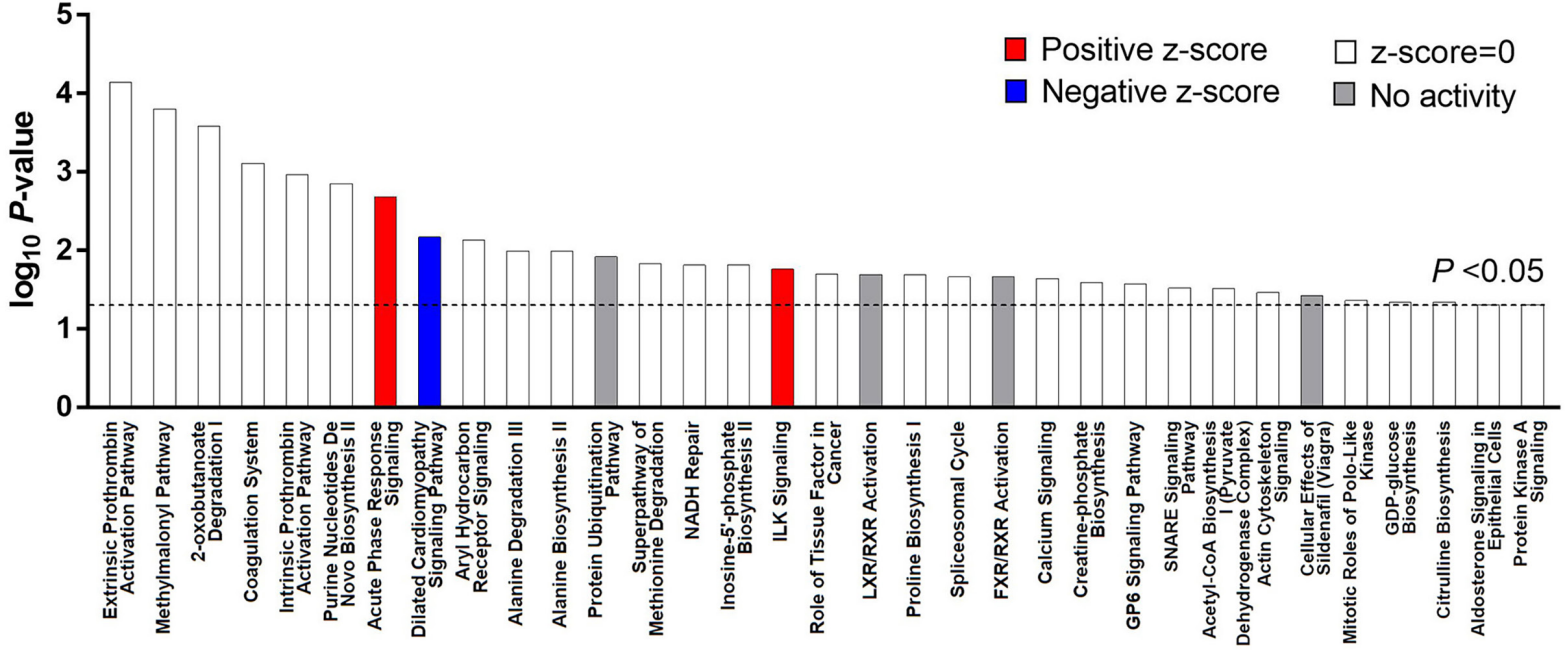
Canonical pathway analysis. The top three canonical pathways were identified according to significant *z*‐value and *p*‐value. Acute phase response signaling (*z* = 1, *p* = .00208), dilated cardiomyopathy signaling pathway (*z* = −2, *p* = .00671), and ILK signaling (*z* = 1, *p* = .0172) were key pathways with highlight associations. *n* = 3.

IPA was used to perform enrichment analysis and investigate the biological significance of the differentially expressed proteins identified between sodium pentobarbital‐treated tumors and control tumors. A total of 33 canonical pathways were first identified based on *p*‐value. Among these pathways, acute phase response signaling (*z* = 1, *p* = .00208), dilated cardiomyopathy signaling pathway (*z* = −2, *p* = .00671), and ILK signaling (*z* = 1, *p* = .0172) were then revealed as key pathways with highlight associations (Figure 3). The details of the abovementioned canonical pathways are shown in Table 3.

**TABLE 3:** Canonical pathway analysis.

Proteins	Protein descriptions	Fold change (Treatment /control)	Expected	Location	Type
Acute phase response signaling (*z* = 1, *p* = .00208)					
FGA	Fibrinogen alpha chain	2.15	Up	Extracellular space	Other
FGB	Fibrinogen beta chain	2.23	Up	Extracellular space	Other
FGG	Fibrinogen gamma chain	1.87	Up	Extracellular space	Other
ITIH4	Inter alpha‐trypsin inhibitor, heavy chain 4	1.81	—	Extracellular space	Other
TAB1	TGF‐beta‐activated kinase 1 and MAP3K7‐binding protein 1	−1.51	Up	Cytoplasm	Enzyme
Dilated cardiomyopathy signaling pathway (*z* = −2, *p* = .00671)					
MYH1	Myosin heavy chain 1	3.75	Down	Plasma membrane	Enzyme
MYH4	Myosin heavy chain 4	3.33	Down	Cytoplasm	Enzyme
MYL1	Myosin light chain 1	3.50	Down	Cytoplasm	Other
TTN	Titin	3.65	Down	Cytoplasm	Kinase
ILK signaling (*z* = 1, *p* = .0172)					
FLNC	Filamin C	2.47	Down	Cytoplasm	Other
MYH1	Myosin heavy chain 1	3.75	Up	Plasma membrane	Enzyme
MYH4	Myosin heavy chain 4	3.33	Up	Cytoplasm	Enzyme
MYL1	Myosin light chain 1	3.50	Up	Cytoplasm	Other

### 3.4 Upstream regulators analysis

IPA analysis revealed that smoothelin‐like 1 (SMTNL1) is an upstream regulator involved in the inhibition of several differentially regulated proteins including FLNC, GAPDH, MYH4, MYL1, and MYOM1 (Table 4). Notably, these proteins are partly consistent with the key proteins in identified canonical pathways dilated cardiomyopathy signaling pathway, and ILK signaling (Table 3). SMTNL1 is a regulatory smooth muscle protein that plays a role in smooth muscle contractility and vascular adaptations related to multiple pathological or physiological processes such as hypertension and pregnancy.^14^ SMTNL1 participates in Ca^2+^ sensitization regulation by inhibition of myosin light chain phosphatase (MLCP) and actin cytoskeleton polymerization via interaction with tropomyosin.^15^ SMTNL1 exerts an inhibitory effect on MLCP related to the phosphorylation of MYL9.^16^ These effects are also modulated by upstream signaling including protein kinase G (PKG) and cyclic guanosine monophosphate (cGMP).^15^, ^17^

**TABLE 4:** Upstream regulators analysis.

Upstream regulator	Molecule type	Predicted activation state	Activation *z*‐score	*p*‐value	Target molecules in the dataset
ESR2	Ligand‐dependent nuclear receptor	—	1.400	.00215	ANK1, CAV1, KRT8, MYH1, THBS4
TP53	Transcription regulator	—	1.265	<.001	ATL3, BICD2, CAV1, CKMT2, GAPDH, HSP90AA1, KPNA2, KRT8, NLRX1, PCCA, PDHB, PLOD2, POLRMT, PPP1R13L, SORBS1, THBS4, TTN
STAT5B	Transcription regulator	—	−0.555	.0197	A1BG, CDK6, DIP2B, MYH1, OBSCN
TAZ	Enzyme	—	−1.342	.0074	CCT6A, CDK6, HSP90AA1, PLOD2, PSMD14
NFE2L2	Transcription regulator	—	−1.982	.136	EIF3E, EIF4G2, HSP90AA1, PSMD14
SMTNL1	Other	Inhibited	−2.236	<.001	FLNC, GAPDH, MYH4, MYL1, MYOM1

**FIGURE 4:**
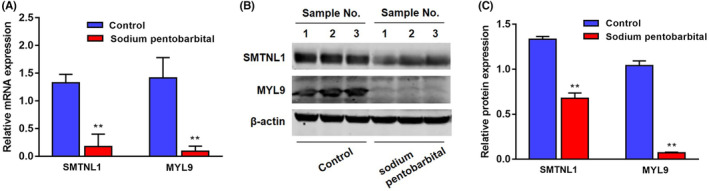
Differential expressions of SMTNL1 and MYL9 in tumor tissues treated with or without pentobarbital. (A) Relative mRNA expressions of SMTNL1 and MYL9 were significantly decreased in pentobarbital‐treated tumor tissues compared with those in controls. (B, C) The protein expressions of SMTNL1 and MYL9 were significantly decreased in pentobarbital‐treated tumor tissues compared with those in controls. *n* = 3, **p < .01 vs. Control.

Due to its pro‐contractive effect on the arteriole smooth muscle, to determine the relationship between SMTNL1 and the anti‐cancer effect of pentobarbital, we did RT–qPCR and Western blotting analyses of SMTNL1 and MYL9 using tumor tissues treated with or without pentobarbital. The relative mRNA expressions of SMTNL1 and MYL9 were found significantly decreased in pentobarbital‐treated tumor tissues compared with those in controls (both *p* < .01, Figure 4A). The protein expressions of SMTNL1 and MYL9 were also significantly decreased in pentobarbital‐treated tumor tissues compared with those in controls (both *p* < .01, Figure 4B,C).

### 3.5 Protein–protein interaction network analysis

**FIGURE 5:**
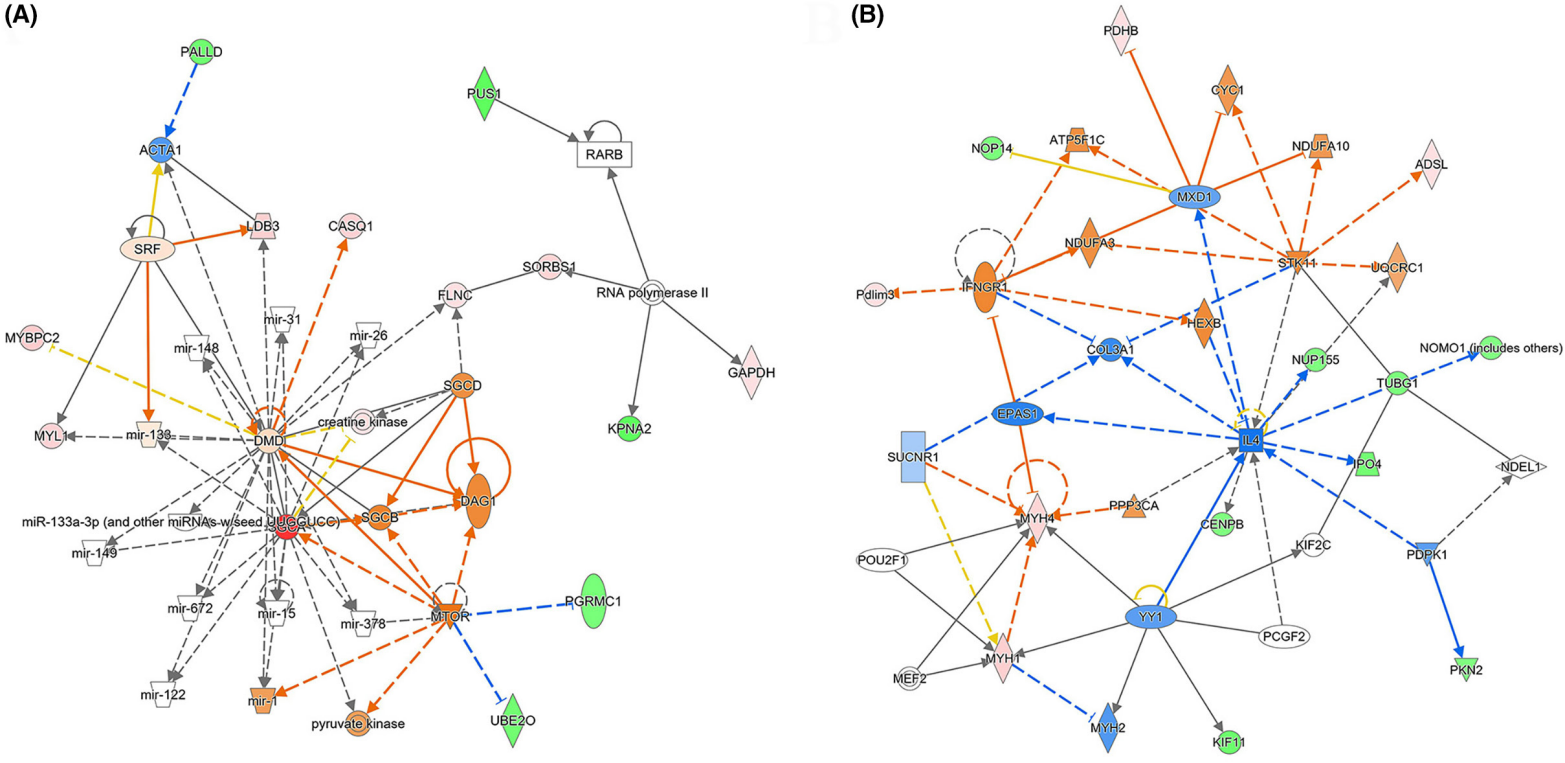
Protein–protein interaction network analysis. Two interaction networks with the key downstream proteins regulated by SMTNL1 are focused. (A) No. 4 network annotation (Table 5) is found to be of developmental disorder, hereditary disorder, organismal injury, and abnormalities with a score of 20 and 13 focus molecules out of the 101 differentially expressed proteins between groups. (B) The top diseases and functions of No. 2 network annotation (Table 5) include organ morphology, organismal injury, and abnormalities, skeletal and muscular disorders, with a score of 20 and 13 focus molecules. *n* = 3.

IPA analysis was performed on the protein–protein interaction networks. A total of 9 significant interaction networks were identified. The network annotations from the IPA analysis of the 101 differentially expressed proteins are listed in Table 5. In these networks, we focused on two interaction networks (No. 4 in Figure 5A and No. 2 in Figure 5B), with the key downstream proteins regulated by SMTNL1. No.4 network annotation is found to be of developmental disorder, hereditary disorder, organismal injury, and abnormalities with a score of 20 and 13 focus molecules out of the 101 differentially expressed proteins between groups (Figure 5A). The top diseases and functions of the No. 2 network annotation include organ morphology, organismal injury and abnormalities, skeletal and muscular disorders, with a score of 20 and 13 focus molecules as well (Figure 5B).

**TABLE 5:** Protein–protein interaction networks analysis.

No.	Molecules in network	Score	Focus molecules	Top diseases and functions
1	ATL3, Atp5k, ATP5MC3, ATP5PB, BICD2, BUB3, CCT2, CCT4, CCT6A, CCT8, CTNNB1, DMAC2L, ETFA, FHL1, HSP90AA1, MYCN, MYOM3, NDUFA11, NDUFA12, NDUFA8, NDUFAF1, NDUFB2, NDUFB6, NDUFC2, NDUFS1, NDUFS6, PLOD2, POLRMT, PPP1R13L, THBS4, TLE3, TP53, TTN, UBQLN4, UQCR10	20	13	Cell signaling, post‐translational modification, protein synthesis
2	ADSL, ATP5F1C, CENPB, COL3A1, CYC1, EPAS1, HEXB, IFNGR1, IL4, IPO4, KIF11, KIF2C, MEF2, MXD1, MYH1, MYH2, MYH4, NDEL1, NDUFA10, NDUFA3, NOMO1 (includes others), NOP14, NUP155, PCGF2, PDHB, Pdlim3, PDPK1, PKN2, POU2F1, PPP3CA, STK11, SUCNR1, TUBG1, UQCRC1, YY1	20	13	Organ morphology, organismal injury and abnormalities, skeletal and muscular disorders
3	ADCYAP1, ATF3, ATP5F1A, BNIP3, Bvht, CFD, CLPP, CREBBP, CTNNBL1, EFEMP1, EFEMP2, EIF3E, EIF4G2, FBLN5, HDAC4, HNF1A, HSPB2, IDH3A, KLHL40, MEF2C, MMP9, MYOM1, MYOT, NFE2L1, NFE2L2, NQO1, PDHB, PSMD14, PTEN, SERPINA10, SETD2, SLC25A11, STOM, TRADD, UGT1A6	20	13	Cardiovascular system development and function, cell‐to‐cell signaling and interaction, cellular compromise
4	ACTA1, CASQ1, creatine kinase, DAG1, DMD, FLNC, GAPDH, KPNA2, LDB3, mir‐1, mir‐122, mir‐133, miR‐133a‐3p (and other miRNAs w/seed UUGGUCC), mir‐148, mir‐149, mir‐15, mir‐26, mir‐31, mir‐378, mir‐672, MTOR, MYBPC2, MYL1, PALLD, PGRMC1, PUS1, pyruvate kinase, RARB, RNA polymerase II, SGCA, SGCB, SGCD, SORBS1, SRF, UBE2O	20	13	Developmental disorders, hereditary disorders, organismal injuries and abnormalities
5	Akt, ALDH4A1, ATP5F1C, Atp5k, calpain, CAV1, CKMT2, CLUH, Cox6c, CPT1B, DYSF, EHD2, EMILIN1, FGB, GNAI3, INPPL1, KRT8, MYO1C, NAA10, NDUFA8, NDUFAB1, NDUFS6, NLRX1, PCCA, PCCB, PDK2, SH2B2, SKIL, SORBS1, TGFB1I1, TKFC, TP53, TRIM72, UQCR10, ZBTB10	20	13	Hematological disease, metabolic disease, organismal injury and abnormalities
6	ALDH18A1, CD14, CDK6, CFB, CGAS, DDX41, DNM2, EGFR, EIF4EBP1, FGA, FGG, GPT, IFNB1, IL1B, ILK, ITGA1, ITIH4, LBP, MED6, miR‐125b‐5p (and other miRNAs w/seed CCCUGAG), mir‐130, mir‐34, MSR1, MYC, NANOG, NCOA2, NOS2, PDK2, PGAM5, PPARG, RCAN1, RCL1, TAB2, Usp17la (includes others), YTHDC1	16	11	Cell death and survival, cell‐to‐cell signaling and interaction, hematological system development and function
7	A1BG, ANK1, APOE, ATM, BCL10, BIRC3, BTK, CASP8, CCL3L3, CCND1, CCND2, CD28, DIP2B, FAS, FOXP3, HLA‐A, ICAM1, IKBKB, IKK (complex), MAP3K14, Map3k7, MAP3K8, NFkB (complex), NFKBIB, NLRX1, NQO1, OBSCN, RCAN1, REL, STAT5B, TAB1, TBCA, TNFRSF1A, TRAF2, TRAF3	9	7	Hematological system development and function, lymphoid tissue structure and development, tissue morphology
8	DIMT1, PHF12	2	1	Cell morphology, cellular assembly and organization, DNA replication, recombination, and repair
9	DHX15, DHX8	2	1	Cellular growth and proliferation, gene expression, RNA post‐transcriptional modification

## 4 DISCUSSION

Compared to non‐TNBC, TNBC displays distinctive malignant behavior, with increased invasiveness and poor prognosis.^18^ Multiple anti‐angiogenic agents have been used to treat TNBC, but limited efficacy is observed and the potential mechanisms are not completely understood.^19^ Current evidence suggests that a vessel normalization strategy is beneficial to the treatment of breast cancer.^20^ To restore the structure and function of tumor vasculature, vessel normalization promotes the perfusion of blood flow and decreases the hypoxia and acidosis in tumor tissues.^21^ Multiple agents, especially antiangiogenic agents, could normalize the tumor vessels and improve the oxygenation and delivery of drugs or immune cells.^22^

The microcirculatory state including hemodynamics and oxygenation plays important role in the malignant behavior of TNBC. We recently revealed that the inhibitory effect of pentobarbital on breast cancer growth is associated with the ability of pentobarbital to normalize the microcirculatory hemodynamics and oxygenation in tumor tissues.^12^ The dose of pentobarbital (50 mg/kg/day) used in vivo in our previous and current studies is the routine dose as an anesthetic agent in mice experiments. Due to the fact that the blood flow perfusion is regulated by vascular tone controlled by smooth muscle cells in the resistance vasculatures,^14^ we hypothesized that key proteins regulating smooth muscle contractility are potentially involved in the antitumor effect of sodium pentobarbital. Furthermore, a comparison of the differentially expressed proteins in breast cancer treated with or without pentobarbital could identify key regulatory proteins in the microcirculatory hemodynamics of tumor tissues. This is a pilot study to determine if this method could be used to reveal new potential targets for tumor microcirculation research.

The current study revealed 101 differentially expressed proteins between groups using LC–MS/MS. We then used the IPA software to analyze the biological functions of the 47 differentially up‐regulated and 54 differentially down‐regulated proteins from pentobarbital‐treated samples. IPA is a web analytical tool that provides meaningful huge protein data on comprehensive regulatory pathways and functional networks.^23^ Firstly, we identified three canonical pathways with highlight associations, including acute phase response signaling, dilated cardiomyopathy signaling pathway, and ILK signaling. The proteins associated with microcirculatory regulation gain more attention in the signaling pathways. Notably, the last two pathways share 3 proteins including MYH1, MYH4, and MYL1 related to smooth muscle contraction. Myosin, composed of heavy chains and light chains, is the important contractile apparatus in smooth muscle cells.^24^ ILK, a kind of focal adhesion protein, mediates multiple downstream signaling associated with breast cancer pathogenesis and could phosphorylate myosin light chain (MYL) to affect smooth muscle contraction.^25^, ^26^

To identify the upstream regulators of these smooth muscle contraction‐related proteins is beneficial to provide a more precise choice in microcirculation targeting therapy for TNBC. We then performed the upstream regulators analysis in pentobarbital‐treated tumor tissues compared to control tumor tissues. The result indicated that SMTNL1 is revealed as an upstream regulator involved in the inhibition of several differentially regulated proteins including FLNC, GAPDH, MYH4, MYL1, and MYOM1. SMTNL1 is a regulatory smooth muscle protein mainly responsible for smooth muscle contractility.^27^ Existing evidence shows that this process involves the interaction of multiple proteins and Ca^2+^ signaling. MYL9 is a myosin regulatory subunit, also known as 20 kDa myosin regulatory light chain (LC20), which plays a critical role in the regulation of smooth muscle cell contractile activity via its phosphorylation.^28^ Increased intracellular Ca^2+^ concentration triggers the subsequent activation of myosin light chain kinase (MLCK) and then the phosphorylation of MYL9 and vasoconstriction.^29^ Conversely, the dephosphorylation of MYL9 is controlled by MLCP, leading to smooth muscle cell relaxation. SMTNL1 regulates cell contraction by promoting Ca^2+^ sensitization via suppressing the effect of MLCP.^15^ Thus, SMTNL1 can inhibit the dephosphorylation of MYL9, resulting in accumulation of phosphorylated MYL9. Furthermore, SMTNL1 can also be phosphorylated by cGMP and then leads to cell vasodilation through Ca^2+^ desensitization.^14^

In the current study, the expressions of SMTNL1 and MYL9 were assayed in pentobarbital‐treated tumor tissues and control tumor tissues. The results indicated that mRNA and protein expressions of both SMTNL1 and MYL9 were significantly decreased in pentobarbital‐treated tumor tissues compared with those in controls. These data suggest that the decreased inhibitory effect of SMTNL1 on MLCP and decreased level of total MYL9 tend to relax the arteriolar smooth muscle and increase the perfusion of capillary networks. This discovery provides an explanation of the role of pentobarbital to normalize the microcirculatory hemodynamics and oxygenation in breast cancer tissues. Furthermore, IPA network analysis of protein–protein interaction reveals 9 significant interaction networks. Of these, two have key downstream proteins regulated by SMTNL1. These highly interconnected networks are more likely to represent significantly correlated biological networks around SMTNL1‐regulated microcirculatory changes in TNBC. In addition, the identified networks contain molecules that are not present in the dataset (101 differentially expressed proteins), providing more meaningful information for regulatory network analysis.

There are some limitations in this study. First, we observed the antitumor effect of sodium pentobarbital in vivo and identified three canonical pathways, but the changes in the identified pathways in MDA–MB‐231 cells in vitro were not assessed in the current study. The molecular mechanisms underlying these observations need to be further investigated. Secondly, SMTNL1 revealed as an upstream regulator, may become a new potential target for tumor microcirculation research. However, the present data are largely correlative. The identified molecules and pathways associated with SMTNL1 and the regulatory effects of these molecules on tumor microcirculation should be deeply explored in future work. Thirdly, we did not prove that inhibition of SMTNL1 is responsible for the anti‐breast cancer effects of sodium pentobarbital through the regulation of tumor microcirculation in tumor tissues. Future studies should further explore the detailed mechanisms through which sodium pentobarbital exerts its anti‐TNBC and microcirculation‐regulatory effects. The determination of the optimal dose of sodium pentobarbital for TNBC inhibition will be important in clinical trials. Further research on SMTNL1 and connecting different biological factors may provide additional therapeutic options for patients with TNBC via targeting tumor microcirculation.

In conclusion, microcirculatory regulation may play an important role in the malignant behavior of TNBC. Key proteins regulating smooth muscle contractility are potentially involved in the antitumor effect of sodium pentobarbital. Using LC–MS/MS and subsequent IPA analysis, we identified three canonical pathways with highlight associations, including acute phase response signaling, dilated cardiomyopathy signaling pathway, and ILK signaling. SMTNL1, revealed as an upstream regulator, is a key protein involved in pentobarbital‐induced growth inhibition signaling in breast cancer. SMTNL1 may become a new potential target for tumor microcirculation research.

## AUTHOR CONTRIBUTIONS

Ailing Li and Jianqun Han participated in the research design. All the authors conducted experiments. Bingwei Li, Ailing Li, and Jianqun Han performed data analysis. Bingwei Li, Ailing Li, and Jianqun Han wrote or contributed to the writing of the manuscript. All the authors read and approved the final manuscript.

## FUNDING INFORMATION

This research was funded by the CAMS Innovation Fund for Medical Sciences (CIFMS) (Grant No. 2022‐I2M‐1‐026), and the Institute of Microcirculation, PUMC Fundamental Research Fund (Grant No. WXH2020‐02).

## CONFLICT OF INTEREST STATEMENT

The authors declare no conflict of interest.

## ETHICS STATEMENT

Animal care and experimental protocols have been approved by the Institutional Animal Care and Use Committee at the Institute of Microcirculation, Chinese Academy of Medical Sciences (China, Approval No. WXH20210109).

## Data Availability

The original contributions presented in this study are included in the article. Further inquiries can be directed to the corresponding authors.
